# IL-17-Mediated Inflammation Promotes Cigarette Smoke-Induced Genomic Instability

**DOI:** 10.3390/cells10051173

**Published:** 2021-05-12

**Authors:** Chao Cao, Baoping Tian, Xinwei Geng, Hongbin Zhou, Zhiwei Xu, Tianwen Lai, Yanping Wu, Zhengqiang Bao, Zhihua Chen, Wen Li, Huahao Shen, Songmin Ying

**Affiliations:** 1 Department of Respiratory and Critical Care Medicine, Second Affiliated Hospital, Zhejiang University School of Medicine, Hangzhou 310000, China; caocdoctor@163.com (C.C.); tianbp@zju.edu.cn (B.T.); geng_xinwei@126.com (X.G.); zhb0401@126.com (H.Z.); zhiweixu@aliyun.com (Z.X.); laitianwen2011@163.com (T.L.); wuyanping@zju.edu.cn (Y.W.); baozhengq@126.com (Z.B.); zhihuachen2010@163.com (Z.C.); liwenzjhz0408@163.com (W.L.); 2 Department of Respiratory Medicine, Ningbo First Hospital, Ningbo 315000, China; 3 Department of Pharmacology, Zhejiang University School of Medicine, Hangzhou 310012, China; 4 Department of Critical Care Medicine, Ningbo Medical Center, Lihuili Eastern Hospital, Ningbo 315100, China; 5 State Key Lab. for Respiratory Diseases, the First Affiliated Hospital, Guangzhou Medical University, Guangzhou 510220, China

**Keywords:** IL-17, inflammation, cigarette smoke, DNA damage response

## Abstract

(1) Background: Chronic inflammation has been regarded as a risk factor for the onset and progression of human cancer, but the critical molecular mechanisms underlying this pathological process have yet to be elucidated. (2) Methods: In this study, we investigated whether interleukin (IL)-17-mediated inflammation was involved in cigarette smoke-induced genomic instability. (3) Results: Higher levels of both IL-17 and the DNA damage response (DDR) were found in the lung tissues of smokers than in those of non-smokers. Similarly, elevated levels of IL-17 and the DDR were observed in mice after cigarette smoke exposure, and a positive correlation was observed between IL-17 expression and the DDR. In line with these observations, the DDR in the mouse lung was diminished in IL-17 KO when exposed to cigarette smoke. Besides this, the treatment of human bronchial epithelium cells with IL-17 led to increased levels of the DDR and chromosome breakage. (4) Conclusions: These results suggest that cigarette smoke induces genomic instability at least partially through IL-17-mediated inflammation, implying that IL-17 could play an important role in the development of lung cancer.

## 1. Introduction

It is well known that chronic obstructive pulmonary disease (COPD) is a significant risk factor for lung cancer [1]. Both COPD and lung cancer are primarily caused by cigarette smoke (CS), and are frequently presented as co-morbidities [1,2]. A better understanding of the relationship between these two diseases could lead to significant advances in the development of new treatments, so the identification of common mechanisms has become a priority in tobacco-related illness research. In a recent study, it was demonstrated that smoking increases the mutational burden, is associated with multiple mutational signatures that contribute to different types of cancer, and increases cancer risk mainly due to the mis-repair of DNA damage caused by tobacco carcinogens [3]. The maintenance of genomic stability is critical for the viability of all organisms. DNA repair plays a fundamental role in the maintenance of genomic stability against the threats posed by both exogenous and endogenous stress. This process prevents the accumulation of DNA damage and its detrimental consequences for chromosomal rearrangements, sensitivity to genotoxins, and cell viability [4]. Nevertheless, thus far, there is only a limited body of data pertaining to the underlying DNA repair mechanisms protecting cells against cigarette smoke (CS)-induced cancers. Moreover, the interplay between DNA damage response (DDR) and inflammation, and the link to cancer, remain largely unexplored. In our previous study, we demonstrated that cigarette smoke exposure promoted interleukin (IL)-17 secretion and upregulated IL-17 expression [5]. IL-17 concentration showed a positive correlation with neutrophil counts in the sputum of COPD patients [6]. Recent studies further showed that IL-17A is essential for small airway fibrosis and inflammation in mice exposed to cigarette smoke, suggesting a role for this cytokine in airway obstruction during COPD [7]. Additionally, we showed that IL-17 increased cigarette smoke-induced lung injury. We therefore performed the present study to explore (i) whether inflammation promotes CS-induced genomic instability, and (ii) whether IL-17 is involved in CS-induced genomic instability.

## 2. Materials and Methods

### 2.1. Patient Samples

Benign lung disease samples were collected for the analysis of CS-induced DDR and airway inflammation. In total, 10 patients with histories of smoking and 6 non-smokers were included in our study. Patients were excluded if they had a previous history of cancer. The study protocol was approved by the Ethics Committee of Ningbo First Hospital (protocol code 2020-R229 and date of approval 15 October 2020).

### 2.2. Cell Culture

Human bronchial epithelial cells (16HBE cells) were purchased from the American Type Culture Collection (ATCC, Manassas, VA, USA) and were cultured in RPMI 1640 (Sigma-Aldrich, St. Louis, MO, USA) supplemented with 10% fetal calf serum. Cells were seeded at 1 × 10^4^ cells/well in 24-well plates and cultured at 37 °C in a humidified atmosphere containing 5% CO_2_/95% air for 24 h before exposure.

### 2.3. Mice

C57BL/6 mice (male, aged 8–10 weeks) were purchased from Slac Laboratory Animal Center (Shanghai, China). IL-17 KO mice (C57BL/6 background) were purchased from the Center for Experimental Medicine and Systems Biology (Institute of Medical Science, University of Tokyo, Tokyo, Japan). Mice were randomly assigned to different treatments (air or cigarette smoke) at the time of purchase to minimize any potential bias. All protocols in this study were approved by the Ethics Committee for Animal Studies of Zhejiang University (protocol code ZJU20170429 and date of approval 28 February 2017), China.

### 2.4. CS Exposure and CS Extract (CSE) Preparation

Mice were exposed to cigarette smoke in a chamber using a smoking machine (Model TE-10, Teague Enterprises, Woodland, CA, USA). The total particulate matter concentrations of the chamber atmosphere were 160–180 mg/m^3^. Mice were exposed for 2 h a day 5 d/week in consecutive 12 or 24 weeks. The control group was exposed to filtered air under identical conditions. To prepare CSE, smoke from 1 cigarette was bubbled slowly through 10 mL of RPMI 1640, which is considered a 100% CSE solution, and then sterilized.

### 2.5. Immunohistochemistry

Immunohistochemical staining was performed on formalin-fixed, paraffin-embedded tissue sections. Briefly, 4 µm-thick sections were deparaffinized in xylene, rehydrated in graded ethanol, and washed twice with PBS. Endogenous peroxidase activity was blocked by incubating sections with 3% hydrogen peroxide in the dark for 10 min. Slides were incubated overnight at 4 °C with the primary antibody (gH2AX, 1:200, Abcam ab26350, Cambridge, MA, USA; IL-17, 1:400, Abcam ab79056, Cambridge, MA, USA). The sections were washed and incubated at room temperature for 1 h with the secondary antibodies. Finally, the slides were exposed to a substrate chromogen mixture and counterstained with hematoxylin and eosin (H&E). Stained slides were analyzed on an Olympus optical microscope and scored according to the number of positively stained cells.

### 2.6. Immunofluorescence

In total, 2 × 10^4^ HBE cells were treated with or without CSE or IL-17 at the indicated concentrations. The cells were washed 3 times with phosphate-buffered saline (PBS), fixed in 4% paraformaldehyde for 30 min at room temperature and permeabilized with 0.2% Triton X-100 for 5 min at 4 °C. After blocking with 5% bovine serum albumin BSA for 30 min, the cells were incubated with primary antibody against gH2AX diluted in PBS containing 5% BSA (1:1000) overnight at 4 °C, washed three times in PBS, and then incubated with a secondary antibody diluted in PBS containing 5% bovine serum albumin for 30 min at room temperature. DNA was counterstained with 1 mg/mL 4′,6-diamidino-2-phenylindole (DAPI) for 10 min at 37 °C. Cells mounted on cover slips were observed with a fluorescence microscope or confocal laser scanning microscope.

### 2.7. Western Blot Analysis

Cells were lysed in RIPA buffer. Equal amounts of protein were loaded onto SDS-polyacrylamide gels (PAGE), fractionated by electrophoresis, and transferred to nitrocellulose membranes (Bio-Rad, Hercules, CA, USA). The membranes were blocked for 1 h with 5% fat-free milk prepared in PBS containing 0.05% Tween-20. Membranes were incubated overnight at 4 °C with gH2AX antibody (Abcam ab26350) or anti-β Actin antibody (Cell Signaling 3700, Boston, MA, USA). Then, membranes were blotted with corresponding secondary antibodies (1:2000 dilution) for 2 h and the protein bands were visualized using enhanced chemiluminescence.

### 2.8. Statistical Analysis

Prism version 5 (GraphPad Software, Inc. La Jolla, CA, USA) and SPSS for Windows, version 13.0 (SPSS Inc., Chicago, IL, USA), were used for data collection and presentation. The data shown are presented as mean ± standard error. Statistical significance was considered at *p* < 0.05 using Student’s t test. Different levels of significance are indicated as * *p* < 0.05, ** *p* < 0.01, *** *p* < 0.001.

## 3. Results

### 3.1. Smoking Induced a DNA Damage Response and Airway Inflammation in Human Tissue

**Figure 1:**
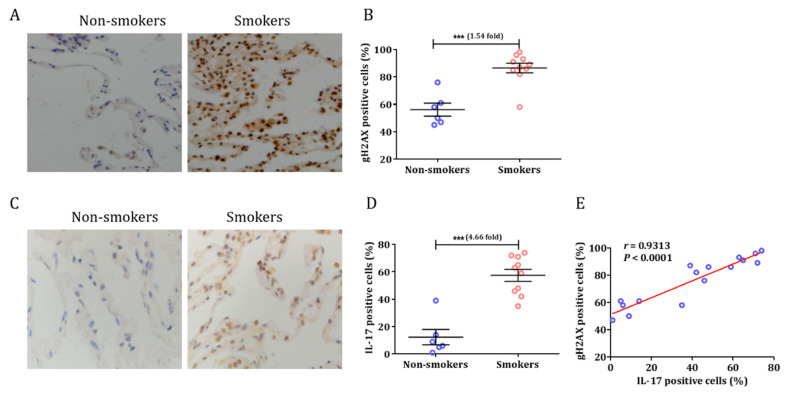
Cigarette smoke induced DDR and airway inflammation in human samples. (**A**) Representative examples of immunohistochemistry of gH2AX expression in the lungs of human specimens. (**B**) Percentages of cells expressing gH2AX in smokers and non-smokers. (**C**) Representative examples of immunohistochemistry of IL-17 expression in human lung tissue. (**D**) Percentages of cells expressing IL-17 in smokers and non-smokers. (**E**) The relationship between IL-17 expression and DDR in lung specimens. (n = 6 mice per group in each experiment. Image magnification of immunohistochemistry: 10 × 100. Data are presented as means ± SEM, *** *p* < 0.001).

The characteristics of patients enrolled in this study are presented in Table S1. We first quantified the degree to which the so-called DDR was associated with smoking, as measured using immunohistochemistry for gH2AX in human lung tissue (Figure 1A). The results showed that the gH2AX expression in the lungs of smokers was 1.54-fold higher than in non-smokers (t = 5.190, *p* = 0.0001; Figure 1B). In our prior study, we demonstrated that Tc17 cells are associated with CS-induced lung inflammation and emphysema [5]. The main function of IL-17 is to coordinate local tissue inflammation via the regulation of pro-inflammatory cytokines [8]. Based on these premises, we investigated the expression of IL-17 in smokers and non-smokers. Interestingly, IL-17 was observed mainly in the lung specimens of smokers (Figure 1C). The expression of IL-17 was significantly increased in the lungs (t = 6.334, *p* < 0.0001; Figure 1D) of smokers compared to those of non-smokers. Moreover, IL-17 expression was strongly correlated with gH2AX (r = 0.9313, *p* < 0.0001; Figure 1E).

### 3.2. Cigarette Smoke Induced a DNA Damage Response in C57BL/6 Mice

**Figure 2:**
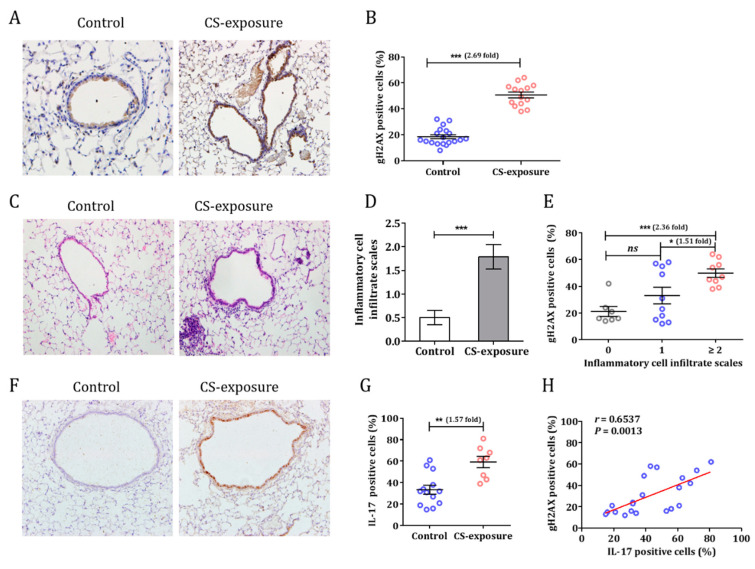
Cigarette smoke induced DDR and airway inflammation in mice. (**A**) C57BL/6 mice were exposed to smoke for 12 weeks and the DDR in lungs was assessed (IHC, gH2AX). (**B**) The percentages of gH2AX-positive cells in control and CS-exposed groups were counted. (**C**) Inflammatory cell infiltration in the lungs of mice after exposure to cigarette smoke. (**D**) Inflammation scores in control and cigarette smoke exposure groups. (**E**) Relationship between inflammation scores and DDR in the mouse lung. (**F**) IL-17 expression in the mouse lung after exposure to cigarette smoke. (**G**) Quantification of IL-17-positive cells in control and cigarette smoke-exposed mice. (**H**) The relationship between IL-17 expression and DNA damage in the lung. (n = 6 mice per group in each experiment. Data are presented as means ± SEM, * *p* < 0.05, ** *p* < 0.01, *** *p* < 0.001).

C57BL/6 mice were exposed to smoke for 12 weeks. A higher level of gH2AX expression in the bronchial epithelial cells of CS-exposed mice than in control mice was observed (50.7 ± 2.5% vs. 18.5 ± 1.5%; *p* < 0.0001), indicating the presence of damaged DNA (Figure 2A,B). In addition, we observed that there was an increase in inflammatory cells infiltrating the airway of CS-exposed mice (Figure 2C). Hematoxylin and eosin (HE) staining revealed that the cellularity of the lungs was disrupted, and the numbers of lymphocytes and neutrophils were increased. Next, we assessed the inflammatory cells infiltrating the lung using previously published criteria (on a 0 to 4+ scale) [9]. The inflammatory score was 1.79 ± 0.26 for the CS group and 0.50 ± 0.15 for controls (Figure 2D). Interestingly, there was a relationship between inflammation scores and DDR in the lung (Figure 2E). A higher IL-17 expression was also observed in CS-exposed mice (Figure 2F,G). Consistent with the findings from human specimens, IL-17 expression was strongly correlated with gH2AX in the lung (r = 0.6537, *p* = 0.0013; Figure 2H).

### 3.3. IL-17 Induced Genomic Instability in Bronchial Epithelial Cells

**Figure 3:**
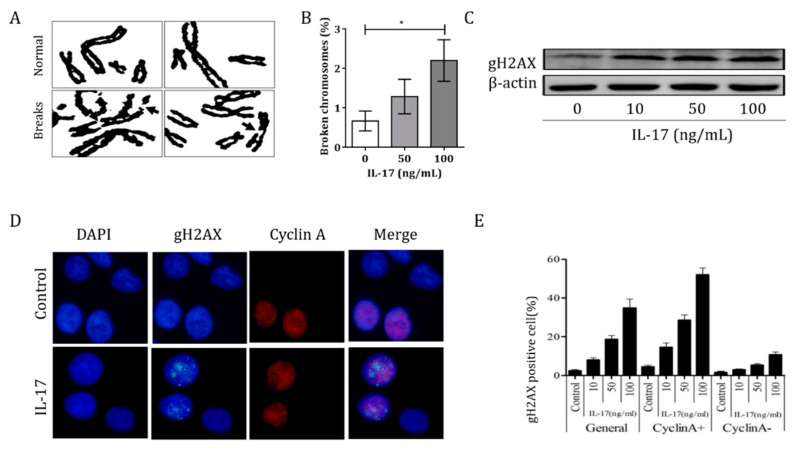
IL-17-induced genomic instability in bronchial epithelial cells. (**A**) Arrows denote broken chromosomes after IL-17 treatment. (**B**) Numbers of broken chromosomes after treatment with increasing IL-17 concentrations. (**C**) Western blot assay for the expression of gH2AX after the treatment of cells with IL-17. (**D**) Representative images of gH2AX and cyclin A staining in control and IL-17 treatment groups (magnification 10 × 100). (**E**) Percentages of gH2AX-positive cells in cyclin A^+^ and cyclin A^-^ cells. (Data are presented as mean ± SEM, * *p* < 0.05).

To assess whether IL-17 can trigger DNA damage and genomic instability, we investigated the chromosome breakages in 16HBE cells after IL-17 treatment (Figure 3A). The fragile site expression was 2.2 ± 0.5% breaks per chromosome, which was higher than that of controls (0.7 ± 0.3%; *p* < 0.05) (Figure 3B). The DNA damage of bronchial epithelial cells by IL-17 was observed in a dose-dependent manner, as showed in Figure S1, and this finding was also confirmed by Western blotting (Figure 3C). We further studied whether the increased DDR following IL-17 treatment was cell cycle-related (Figure 3D). In the 24 h period after IL-17 treatment, the majority of gH2AX foci were observed in cyclin A-positive cells and were therefore in the S/G2 phase (Figure 3E), which indicates that IL-17 treatment induces cell cycle-related DDR in 16HBE cells. In order to identify the genes involved in IL-17-induced genomic instability, we performed an RNA-seq experiment. The RNA was isolated from 16HBE cells after treatment with or without IL-17 for 24 h. The RNA samples were reverse-transcribed into cDNA and cDNA libraries were constructed using the mRNA-sequencing assay. Based on the expression levels of known genes, we identified 583 up- and 704 downregulated genes. Several genes specifically related to the DDR were identified in IL-17-induced genomic instability in bronchial epithelial cells (Table S2).

### 3.4. CS Induced Airway Inflammation and DDR in IL-17 KO Mice

**Figure 4:**
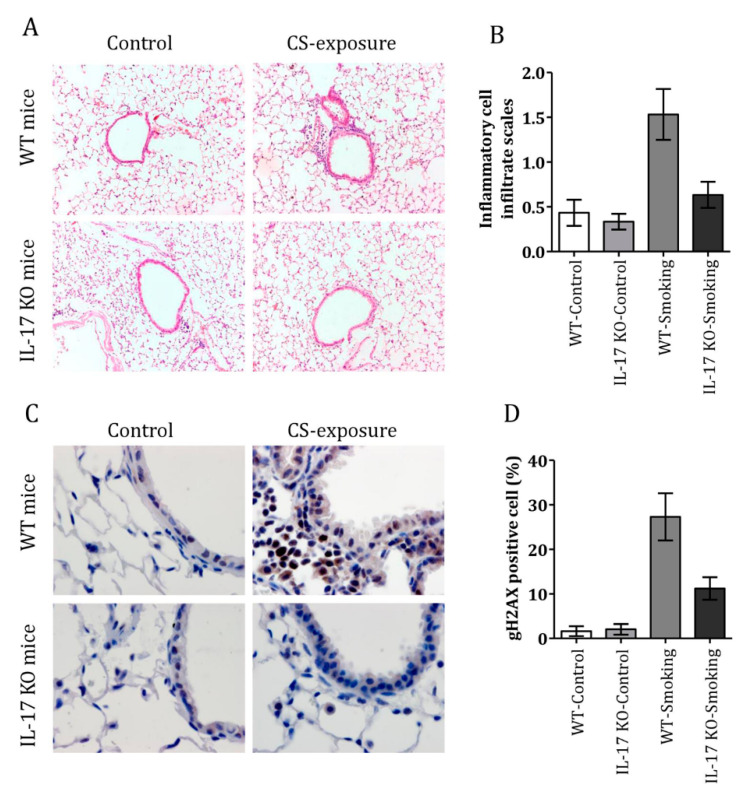
Cigarette smoke induced airway inflammation and a DNA damage response in IL-17 KO mice. (**A**) H&E staining of lung tissue in IL-17 KO and C57BL/6 mice with or without cigarette smoke exposure (magnification 10 × 40). (**B**) Inflammation scores in the lungs of control and cigarette smoke-exposed mice. (**C**) Representative examples of gH2AX-positive cells in the lungs of IL-17 KO and control mice with or without cigarette smoke exposure (magnification 10 × 100). (**D**) Percentages of gH2AX-positive cells in the lungs of IL-17KO and control mice with or without cigarette smoke exposure. (n = 6 mice per group in each experiment. Data are presented as mean ± SEM).

IL-17 knockout (KO) mice and C57BL/6 mice were exposed to smoke for 12 weeks. The inflammation scores of IL-17 KO and C57BL/6 mice in the CS-treated group were 0.63 ± 0.15 and 1.53 ± 0.28, respectively (Figure 4A, B). In addition, the DDR was decreased in IL-17 KO mice when exposed to CS (Figure 4C), and fewer gH2AX-positive cells were observed in IL-17 KO compared to C57BL/6 after CS-exposure (27.3 ± 5.3% vs. 1.2 ± 2.5%; *p* = 0.02) (Figure 4D). These findings further demonstrate that IL-17 increases CS-induced airway inflammation, and is involved in CS-induced genomic instability.

## 4. Discussion

It is well established that CS is a major preventable risk factor for many diseases, including cancer and heart disease. The mechanisms by which CS leads to disease, however, are still not fully understood, but it is known that smoke exposure triggers cellular DNA damage, causing the accumulation of somatic cell mutations, which contribute to disease development. The current study demonstrated that CS-exposure induces DDR in vitro (in HBE cells) and in vivo (in the lung tissue of both mice and humans), suggesting an increased genomic instability. In addition, we observed that IL-17-mediated inflammation promoted this CS-induced genomic instability. These data are the first to suggest a link between IL-17, CS-induced DDR and genomic instability.

In the present study, we found that CS exposure induced DDR in the lung, which could be explained by the pro-inflammatory effect of CS. H&E staining revealed a large number of infiltrated inflammatory cells in the lungs of CS-exposed mice, and an increase in IL-17 expression. The main function of IL-17 is to coordinate local tissue inflammation via the regulation of pro-inflammatory cytokines [8]. Our study showed that IL-17 induced, and was positively correlated with, the DDR and genomic instability of HBE cells, and its knock-out led to both reduced airway inflammation and a reduced DDR in the lungs of mice after CS exposure. This suggests that IL-17-mediated inflammation promotes CS-induced DDR.

We found it surprising that there was a positive correlation between IL-17 expression and the DDR. The increase in IL-17 may be a critical process in the bioactivation of the CS-induced DDR in the lung. We found that IL-17 triggered the DDR and chromosome breakages in vitro. After IL-17 addition, the majority of gH2AX foci were observed in cells expressing cyclin A, which indicates that the increased DDR seen with IL-17 was due to cell cycle arrest.

COPD is a chronic airway inflammatory disease and tobacco smoke is by far the most important risk factor for COPD worldwide [10]. It is reported that smoking causes inflammatory responses in the lungs and leads to the characteristic pathological lesions of COPD [11]. Patients with COPD are at a greater risk of lung cancer than smokers without COPD with any level of tobacco exposure [12]. An increased DDR in circulating white blood cells has been observed in smokers and COPD patients [13,14,15], providing evidence for widespread systemic inflammatory effects and confirming the pro-inflammatory effects of smoking. Interestingly, inhaled corticosteroids reduce local and systemic inflammation among patients with COPD [16]. Additionally, inhaled corticosteroids may have a potential role in lung cancer prevention in patients with COPD [17].

Our data show that IL-17 mediated CS-induced DDR, which may contribute to the genesis of lung cancer. Regardless, the role of IL-17 in cancer remains controversial. Emerging evidence shows that IL-17 can support tumor growth, tumor progression, and metastasis [18,19,20].

The results from our study take us a step closer to understanding how smoke exposure causes disease, including lung cancer and COPD. CS contains a large number of chemical compounds, many of which can directly cause DNA damage and genomic instability. In response to DNA damage, cells either repair the damage or activate pathways leading to programmed cell death so as to maintain genome integrity [21]. The potency of cigarettes may result from their ability to induce DNA damage while failing to trigger apoptosis, which results in epigenetic changes or somatic cell mutations potentially leading to the development of disease [22].

## 5. Conclusions

In summary, we identified IL-17 as a link between inflammation and genomic instability. Our study results suggest that the CS-induced DDR in the lung is due to its pro-inflammatory effect. CS exposure was found to promote IL-17 expression and induce substantial inflammatory cell infiltration in the mouse lung. Secondly, we showed that IL-17 directly induced DDR and genomic instability in HBE cells, and that CS-induced DDR and inflammatory cell infiltration into the lung were decreased in IL-17 KO mice. Together, these results suggest for the first time that IL-17 promotes CS-induced airway inflammation, DDR and genomic instability, and imply that the targeting of IL-17 could be a promising approach for the treatment of CS-related diseases.

## Data Availability

All data generated or analyzed during this study are included either in this article or in the supplementary information files.

## Supplementary Materials

The following are available online at https://www.mdpi.com/article/10.3390/cells10051173/s1, Figure S1: IL-17-induced genomic instability of bronchial epithelial cells. 16HBE cells were seeded into 24-well plates and were exposed to increasing concentrations of IL-17 for 24 h. (A) Representative images of gH2AX staining in HBE cells. (B) Percentages of gH2AX-positive cells in the control and CS exposure group with increasing concentrations of IL-17, Table S1: Clinical characteristics of recruited participants, Table S2: Related to DNA damage response genes was identified by RNA-seq.
