# Supplementary material for: IL-17-Mediated Inflammation Promotes Cigarette Smoke-Induced Genomic Instability
Source: Cells. 2021 May 12;10(5):1173. doi: 10.3390/cells10051173 (PMC8151076; doi:10.3390/cells10051173)
Supplement: Supplementary file 1 [file cells-10-01173-s001.zip › cells-1157115-supplymentary final.pdf]

cells MDPI

# Supplementary

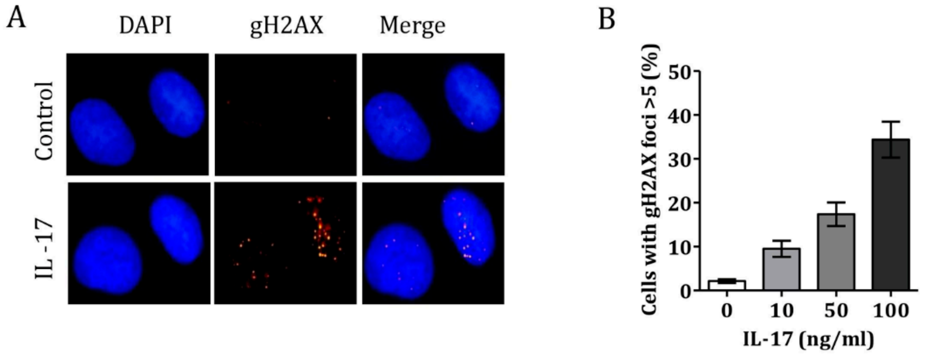


**Figure S1.** IL-17 induced genomic instability of bronchial epithelial cells. 16HBE cells were seeded into 24-well plates and were exposed to increasing concentrations of IL-17 for 24 h. (A) Representative images of gH2AX staining in HBE cells. (B) Percentages of gH2AX positive cells in control and CS-exposure group with the increasing concentrations of IL-17.

**Table S1.** BMI, body-mass index; FEV1/L, one-second forced expiratory volume; FEV1 (%), predicted one-second forced expiratory volume; FVC/L, forced vital capacity; FVC (%), predicted forced vital capacity; FEV1/FVC, forced expiratory volume in 1 second (FEV1)/forced vital capacity (FVC) ratio.

**Table S1.** Clinical characteristics of recruited participants.

| | Smokers (n = 10) | Non-Smokers (n = 6) | *P* Value |
|---|---|---|---|
| Age, years | | | 0.355 |
| Mean ± SD | 50.40 ± 8.00 | 54.83 ± 10.52 | |
| BMI | 23.98 ± 1.92 | 23.31 ± 2.14 | 0.537 |
| Gender, n (%) | | | 0.518 |
| Male | 9 (69.20%) | 4 (30.80%) | |
| Female | 1 (33.30%) | 2 (66.70%) | |
| Smoking index | | | |
| Pack-years | 29.8 ± 13.82 | | |
| Lung function | | | |
| FEV1/L | 3.16 ± 0.60 | 2.98 ± 0.81 | 0.626 |
| FEV1 (%) | 96.50 ± 14.09 | 97.02 ± 16.75 | 0.948 |
| FVC/L | 3.98 ± 0.62 | 3.48 ± 0.74 | 0.164 |
| FVC (%) | 99.83 ± 9.64 | 96.33 ± 12.23 | 0.535 |
| FEV1/FVC | 78.87 ± 3.55 | 82.19 ± 7.93 | 0.264 |

**Table S2.** Sequencing of total RNA from 16HBE cells treated with IL-17 or medium was performed in Zhejiang University School of Medicine (Hangzhou, China). Based on the quantitative results of expression levels, the genes with differential expression between two groups of Low and High concentrations were analyzed. The difference analysis software was edgeR, and the screening threshold was |log2FC = ≥ 1 & p adjust < 0.05. The free online platform was used for the data analysis.

**Table S2.** Related to DNA damage response genes were identified by RNA-seq.

| Functions | Genes |
|---|---|
| DNA replication | CDC25A, TERT, RRM2, LIG4 |
| Postreplication repair | UBA7, RAD18, USP1, UBE2L6, POLD4 |
| DNA replication initation | MCM10, CCNE1, CCNE2, POLD4 |
| DNA replication checkpoint | NAE1 |
| Regulation of DNA replication | AREG, TIPIN, EREG, IGF1R, TP53, IL6, CSF2, JUN, GTPBP4, CDC6, PML, ID3 |
